# Imaging Cytometry of Human Leukocytes with Third Harmonic Generation Microscopy

**DOI:** 10.1038/srep37210

**Published:** 2016-11-15

**Authors:** Cheng-Ham Wu, Tzung-Dau Wang, Chia-Hung Hsieh, Shih-Hung Huang, Jong-Wei Lin, Szu-Chun Hsu, Hau-Tieng Wu, Yao-Ming Wu, Tzu-Ming Liu

**Affiliations:** 1 Institute of Biomedical Engineering, National Taiwan University, Taipei 10617, Taiwan; 2 Cardiovascular Center and Division of Cardiology, Department of Internal Medicine, National Taiwan University Hospital and College of Medicine, Taipei 10002, Taiwan; 3 Department of Laboratory Medicine, National Taiwan University Hospital, Taipei 10002, Taiwan; 4 Department of Mathematics, University of Toronto, Toronto, Canada; 5 Department of Surgery, National Taiwan University Hospital and College of Medicine, Taipei 10002, Taiwan; 6 Molecular Imaging Center, National Taiwan University, Taipei 10617, Taiwan; 7 Faculty of Health Sciences, University of Macau, Macao SAR, China

## Abstract

Based on third-harmonic-generation (THG) microscopy and a k-means clustering algorithm, we developed a label-free imaging cytometry method to differentiate and determine the types of human leukocytes. According to the size and average intensity of cells in THG images, in a two-dimensional scatter plot, the neutrophils, monocytes, and lymphocytes in peripheral blood samples from healthy volunteers were clustered into three differentiable groups. Using these features in THG images, we could count the number of each of the three leukocyte types both *in vitro* and *in vivo*. The THG imaging-based counting results agreed well with conventional blood count results. In the future, we believe that the combination of this THG microscopy-based imaging cytometry approach with advanced texture analysis of sub-cellular features can differentiate and count more types of blood cells with smaller quantities of blood.

Leukocytes also called white blood cells (WBCs), play important roles in the immunity of humans. In peripheral blood, neutrophils, monocytes, and lymphocytes are three major types of circulating WBCs. Eosinophils and basophils, although relatively scarce, are also measured in routine clinical practice. For a healthy individual, the percentages of neutrophils, monocytes and lymphocytes are 50–70%, <10% and 25–33%, respectively. Different immune challenges can change the percentages and the number densities. Physicians often rely on such quantitative information to make differential diagnoses. For example, bacterial infections often cause an increased neutrophil count, while viral infections and auto-immune disorders raise lymphocyte counts. Conventionally, to classify leukocytes, a blood smear was prepared and stained with a special stain. Then, the technician can differentiate the white blood cells and make a classification report. However, for routine blood tests, this procedure is time-consuming, and the sampling error could be large. With the invention of flow cytometry, the cell counting and differentiation speeds were greatly accelerated. By measuring the forward scatter (dependent on cell size) and side scatter (dependent on cell granularity) intensities of leukocytes, the three major types of WBCs could be classified into three distinct groups in the scatter plot. With the help of depolarized side scatter measurements, eosinophils can further be distinguished from neutrophils^1^. The counting of WBC differentials can thus be performed efficiently without labeling^2^.

However, the aggregation of platelets or abnormal leukocytes sometimes causes cytometric analysis to fail. In that situation, manual review under a microscope is still necessary to confirm the diagnosis. Therefore, in the workflow of WBC counts, imaging cytometry of blood films is still required as a quality check. To integrate this task into the flow cytometry and accelerate the manual review, the imaging flow cytometer has been developed. It not only records scattering and fluorescence properties, but it also acquires corresponding microscopic images of the cells in a high throughput fashion^3^. The morphological and textural features of images can be used to distinguish dead cells^4^,^5^, rare cells, and circulating tumor cells^6^ from large backgrounds of leukocytes and platelets. Combined with fluorescent labels, imaging flow cytometry can also analyze the cellular uptake of chemicals and drugs^7^,^8^. So far, most imaging flow cytometry work requires blood sampling and labeling to observe the cell morphology *ex vivo*. Limited by poor imaging resolution and contrast, label-free and non-invasive *in vivo* WBC differentiation has not been available yet. Recently, beneath the human skin, we made the first step toward this goal by demonstrating *in vivo* imaging flow cytometry of blood cells in humans^9^. Without labeling, the granularity of leukocytes can be reflected by third harmonic generation (THG) contrast^10^. Compared with other label-free modalities of leukocyte imaging, such as reflectance confocal microscopy^11^,^12^, micro-optical coherence tomography (μ-OCT)^13^, and spectrally encoded confocal microscopy^14^, the THG contrast of our method is background-free. Moreover, due to the nonlinear nature of the signals, it has better *in vivo* resolution to visualize nuclei and sub-cellular details^9^,^10^. Besides, the THG signals can be resonantly enhanced by the material absorption^15^,^16^. Coincidentally, the second overtone absorption band of R-CH_2_-R stretching modes peaks around 1210 nm^17^. When excited at 1150–1250 nm, this characteristic absorption band of lipids can enhance the THG yields^18^ and make THG microscopy an imaging modality sensitive to lipids^19^. These imaging features are critical for the differentiation of WBC subtypes. Nevertheless, without antibody labeling, we did not know exactly the identities of cells from the captured images of *in vivo* imaging flow cytometry. We need to isolate leukocytes of certain types and acquire their *ex vivo* ground-truth images as a learning data set for feature extraction and the development of a machine-learning algorithm.

In this report, we made further steps to ensure the THG imaging cytometry of human leukocytes has differentiable features for the three major types of WBCs. Simply using the average THG intensity within cells and the cell size estimated from the cross-sectional area, we confirmed in the scatter plot that the distribution of each type was clustered and separated from others. They can be easily differentiated with a straightforward k-means clustering algorithm. This quality of separation is valid for different volunteers at different sampling time-points. Exploiting the threshold learned from those data, we differentiated and counted the number of WBCs in a whole-blood sample. Both the percentages and number densities agreed well with the results from clinical laboratory examination. Furthermore, we found these THG features can also differentiate circulating human leukocytes *in vivo*. With these ground-truth images and differentiable features, combined with texture analyses and machine-learning algorithms, we believe that *in vivo* WBC differentiation and automatic counting can be achieved in the near future.

## Results

### Obtaining ground-truth THG images of isolated leukocytes

**Figure 1:**
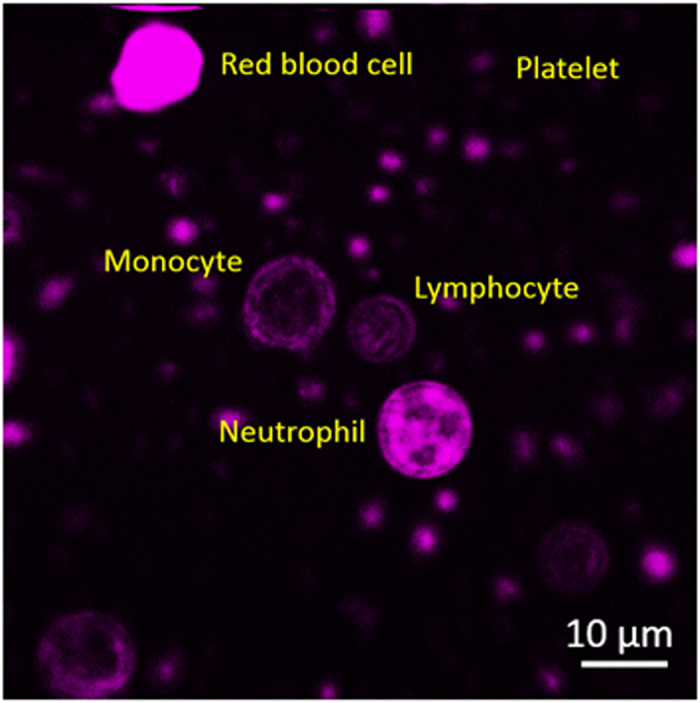
THG sectioning image of a whole blood smear at 1-hour post blood sampling. Different types of leukocytes could be distinguished by their size, THG intensity, and nuclear morphology. Field of view: 73 × 73 μm.

To acquire ground-truth THG images of leukocytes for *in vivo* imaging flow cytometry, WBCs need to be isolated with the least perturbation to their physiological properties right after blood sampling. Environmental stimulation may induce changes in cell size, morphology, and granularity. Therefore, the effects of every manual operation shall be investigated. Right after blood sampling, the first potential stimulus is the anticoagulant. In our procedure, whole blood was collected with ethylenediamine tetraacetic acid (EDTA)-coated tubes. Compared with hematologic examination using standard Liu’s stain (Fig. S1*a*), THG sectioning images of red blood cells (RBCs), polymorphonuclear neutrophils, monocytes, smaller lymphocytes, and scattered platelets can be clearly identified in a blood smear (Fig. 1). Their nuclear morphology (dark contrasts) and size resemble those under Liu’s stain. In contrast, we found most leukocytes collected by heparin tubes were deformed and inadequate for further investigation (Fig. S2).

The second factor that may alter the morphology of cells is a contact of leukocytes with the surface of the container. When leukocytes settle down, they may stick to the surface of the container and generate granules due to stress from the environment. We observed that leukocytes settled down for more than 4 hours at room temperature and their THG images showed irregular shapes and bright granules (Fig. S3). To avoid this effect, we took THG images of leukocytes right after loading them into the counting chamber and finished the measurement within one hour.

**Figure 2:**
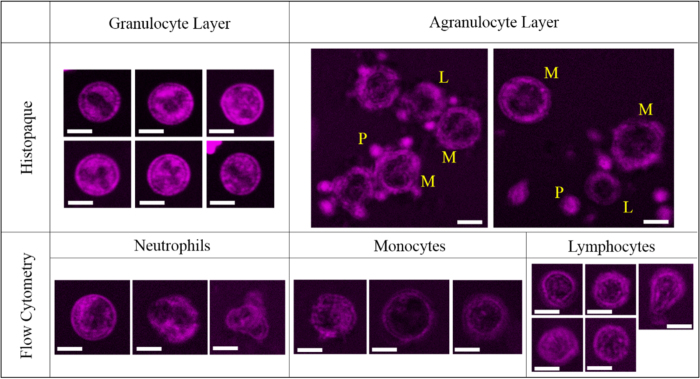
THG sectioning images (magenta) of various leukocytes isolated with Histopaque gradient centrifugation (upper row) and with flow cytometry (bottom row). Scale bars: 5 μm. L: lymphocyte; M: monocyte; P: platelet.

**Figure 3:**
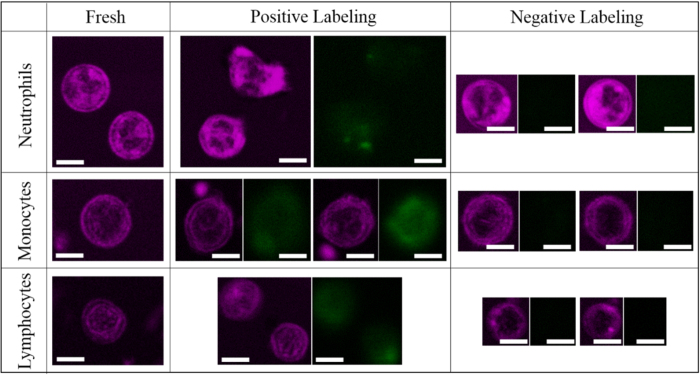
THG sectioning images (magenta) of various leukocytes in a blood smear, positive-labeled and negative-labeled cells. Target cells emit FITC fluorescence (green) in the positive-labeled group, but they lack fluorescence in the negative-stained group. Scale bars: 5 μm.

The third factor would be the WBC isolation procedure. To collect particular types of WBCs for the subsequent analysis, we first used gradient centrifugation with Histopaque-1119 and -1077. The granulocytes and agranulocytes can be separated due to their difference in buoyant density. As verified with Liu’s stain, almost all of the leukocytes in the granulocyte band were neutrophils, and their THG morphology did not change markedly (Fig. S1*b*). Similarly, the morphology of monocytes and lymphocytes in the agranulocyte band was not affected (Fig. S1*c*). However, there were platelets attached to the cell surfaces, which may affect the analysis in imaging cytometry. To increase the purity of isolation, we further sorted the cells with a flow cytometer. When gated with forward scatter and side scatter, three major types of WBCs were sorted and isolated. The THG morphology of the monocytes and lymphocytes did not change much after this process, while most of the granulocytes were distorted somehow (Fig. 2). As a result, ground-truth THG images of neutrophils should be isolated from the granulocyte band of a Histopaque centrifugation gradient. For the isolation of monocytes and lymphocytes, we further added an immunofluorescence label with anti-FITC antibody into the sorting process. Obviously, the cell morphology was not affected either by positive labeling of target cells or negative labeling of other non-target cells (Fig. 3). Morphological changes only occurred in granulocytes. Finally, ground-truth THG images of lymphocytes and monocytes should use cells purified by flow cytometry with negative labeling. After ensuring the least disruptive method of WBC isolation, we drew blood from three volunteers to measure ground-truth THG images of various leukocytes. For the reduction of statistical error, more than 30 cells were imaged for each type of leukocyte.

### The differentiation of isolated WBCs with THG images

**Figure 4:**
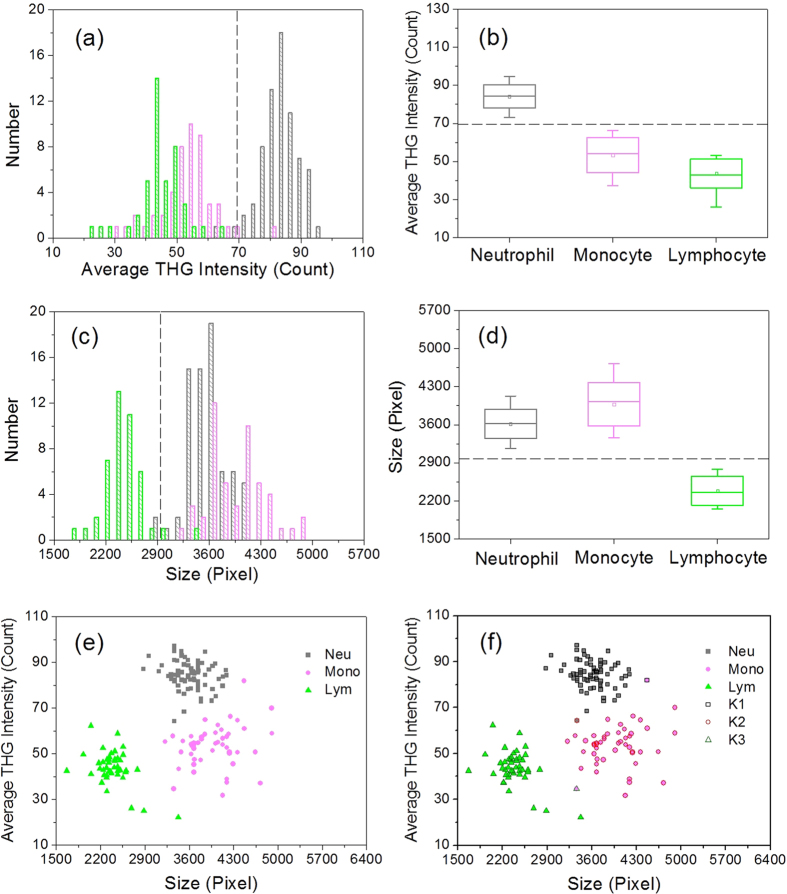
(**a**,**c**) Histograms, (**b**,**d**) box charts, and (**e**,**f**) scatter plots of (**a**,**b**) the average THG intensity and (**c**,**d**) size of WBCs. The neutrophils (gray), monocytes (light magenta), and lymphocytes (green) were collected from the same tube of blood drawn from the same volunteer. In the box chart, the box range covers the mean ± standard deviation, and the whiskers cover 5% to 95% of the data. Three groups of data, K1 (black open square), K2 (open red circle), and K3 (open olive triangle), were clustered by the k-means clustering analysis. Neu: neutrophils; Mono: monocytes; Lym: lymphocytes.

After segmentation at the cell’s boundary, we analyzed the average THG intensity within the cell and the cell’s size as differentiable features. Cell size was approximated as the number of pixels enclosed within the cell boundary at the sectioning plane. The average THG intensity was calculated over these enclosed pixels. Applying histogram analyses on WBCs from the same volunteer (Fig. 4), we found that the THG intensity of granular neutrophils (gray bars, 84.2 ± 6.1) was obviously higher than that of agranular monocytes (light magenta bars, 53.3 ± 9.1) or lymphocytes (green bars, 43.7 ± 7.5). Considering a 5–95% population range (whiskers in the box chart of Fig. 4b), a threshold level of 70 (dashed line in Fig. 4a,b) was identified for differentiation. This result validated that THG intensity can reflect the granularity of human leukocytes and serve as a significant differentiation feature for WBCs. In the size analysis (Fig. 4c), lymphocytes obviously have smaller enclosed pixel numbers (green bars, 2387 ± 267) than neutrophils (gray bars, 3618 ± 269) and monocytes (light magenta bars, 3976 ± 398). A size threshold of 2956 pixels is able to clearly distinguish lymphocytes from the other two cell types (Fig. 4d). Putting all data points in a THG intensity/size scatter plot, it is obvious that the three major types of WBCs can be distinguished from each other (Fig. 4e). To make the consistent and robust differentiation, we applied a k-means clustering algorithm^20^ to the data points of all WBCs. The three calculated centroids [(84.4,3632), (53.3,3965), (43.5,2410)] agree well with the mean values of neutrophils (84.2,3618), monocytes (53.3,3976), and lymphocytes (43.7,2387). Analyzing the ground-truth THG images of leukocytes, the sensitivity and specificity of differentiation are above 96% and 99%, respectively (Time point T1 of Volunteer V1 in Table S1).

**Figure 5:**
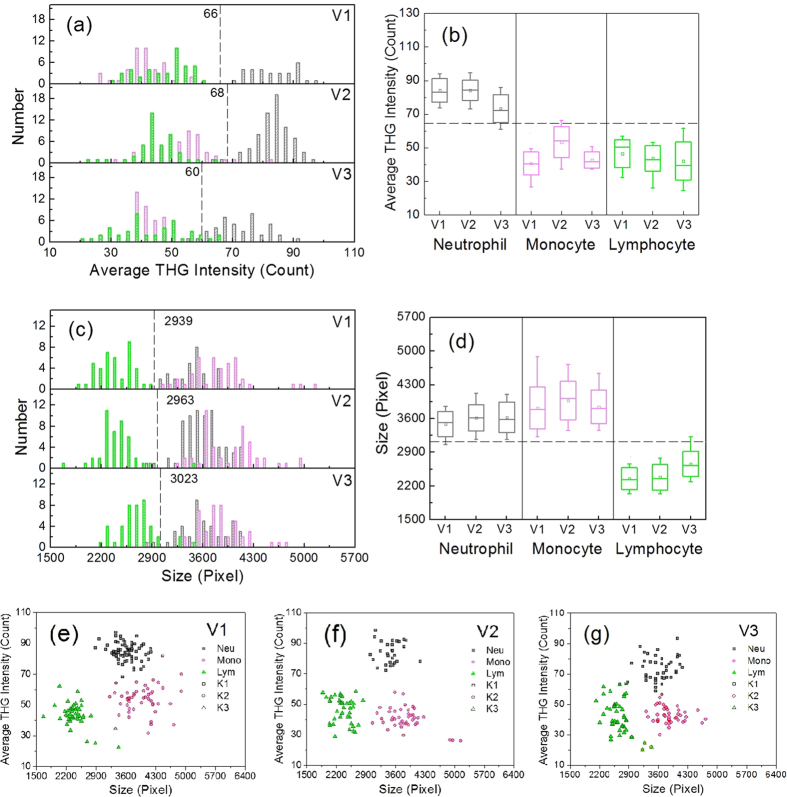
(**a**,**c**) Histograms, (**b**,**d**) box charts, and (**e**,**f**) scatter plots of (**a**,**b**) the average THG intensity and (**c**,**d**) size of WBCs. The neutrophils (gray), monocytes (light magenta), and lymphocytes (green) were collected from three different volunteers, V1, V2, and V3. In the box chart, the box range covers the mean ± standard deviation and the whiskers cover 5% to 95% of the data. Three groups of data, K1 (black open square), K2 (open red circle), and K3 (open olive triangle), were clustered by the k-means clustering analysis. Neu: neutrophils; Mono: monocytes; Lym: lymphocytes.

From the same volunteer, we also sampled his peripheral blood at two other time points, T2 and T3, separated by several days. The THG intensity of neutrophils was relatively lower but still higher than that of leukocytes without significant cytoplasmic granules (Fig. S4a and S4*b*). Lymphocytes were distinguishable from the other two types of WBCs by their size (Fig. S4*d*). Three separable clusters can still be recognized on the scatter plots (Fig. S4*f* and S4*g*). The corresponding differentiation sensitivity and specificity for all types of WBCs were 93–98% and 97–100%, respectively (Table S1). Therefore, for a healthy volunteer, the time point of blood sampling would not affect the differentiation sensitivity and specificity. This was also the case for a second volunteer (Fig. S5 and Table S1). Only at the second time point, some small-sized neutrophils (Fig. S5*b*) reduced the sensitivity of detection to 80%. To understand the individual variation of WBC differentiation, we further recruited the third volunteer and conducted a volunteer-dependent analysis (Fig. 5).

We found that, by considering a 5–95% distribution, there is no universal threshold of THG intensity or size for WBC differentiation (Fig. 5b,d). WBC types need to be differentiated on two-dimensional THG intensity/size scatter plots (Fig. 5e–g). After the application of the k-means clustering algorithm, the average differentiation sensitivity/specificity of our THG imaging cytometry method was 96%/99%, 98%/97%, and 97%/99% for neutrophils, monocytes, and lymphocytes, respectively. By putting together all the data points from three volunteers, interestingly, we found that box charts of THG intensity and size can have a clear-cut threshold at 63 and 3050, respectively (Fig. S6). In the combined scatter plot, the feature of the three clusters remained. The differentiation sensitivity and specificity of the k-means clustering algorithm for each type of WBCs were above 95% and 97%, respectively. These statistical results support the integration of two THG imaging features and a basic k-means clustering algorithm to differentiate the three major WBC subtypes from any healthy individual at any sampling time point. Before closing this section, we mention that textural features of cell images could be included to further distinguish different cell types. For example, under the rotational invariance assumption of texture features, a texture feature is determined by the co-occurrence matrix for pairs of adjacent horizontal pixels. A preliminary study shows that texture features, such as correlation, have the potential to increase the overall clustering accuracy (See Fig. S8). To fully utilize these features, we could apply the modern machine learning technique to further explore the underlying nonlinear structure. A systematic study of these features and the nonlinear techniques are out of the scope of this paper, and the results will be reported in future work.

### Counting of WBCs in whole blood with THG images

With the proof of concept on isolated leukocytes, we next move forward to validate the capability of our method in the clinical scenario; that is, the identification, differentiation, and counting of WBCs in whole blood. We recruited five volunteers and sampled their blood into a 6-mL tube. Half of the blood was sent to the department of laboratory medicine in NTUH for a complete blood count (CBC) with WBC differential. The other half was diluted 10 times with phosphate-buffered saline (PBS) and then loaded into a homemade counting chamber for THG imaging. The loaded chamber was then mounted on the microscope platform. By moving the focal plane of the THG imaging into the chamber region, we could observe the blood cells sandwiched between cover glasses. To obtain large enough numbers of WBCs, we transversely scanned through a 2 × 2 mm^2^ area within each chamber. Because the water-glass interface has a strong THG signal, when we translate the objective toward the chamber, we can clearly identify those two interfaces of these materials in the counting chamber and measure the axial distance between them. As shown in Fig. 1, WBCs can be easily identified because the red blood cells have a much stronger THG signal and the platelets have much smaller size. Considering the area scanned and the thickness of the chamber, we calculated the volume of the blood under imaging and then the number density of WBCs. The WBC densities of the five volunteers fall within the physiological range of 4000–11000 per microliter. Applying the threshold determined in Fig. 5, the percentage of neutrophils, monocytes and lymphocytes in healthy volunteers were approximately 52–70%, 3–14% and 22–34%, respectively (Table 1). The percentages of WBC types from counting were close to and showed a good correlation (r = 0.8863, p < 0.05) with the CBC results. Regarding the absolute density of the total WBCs, there was 20% variation, which might be caused by PBS evaporation or space compression between the two cover glasses over time, leading to higher cell density in some cases. We think these errors can be greatly reduced by replacing this sample holder with reliable counting chambers designed for short working-distance objectives.

### Counting of WBCs from *in vivo* THG images

**Figure 6:**
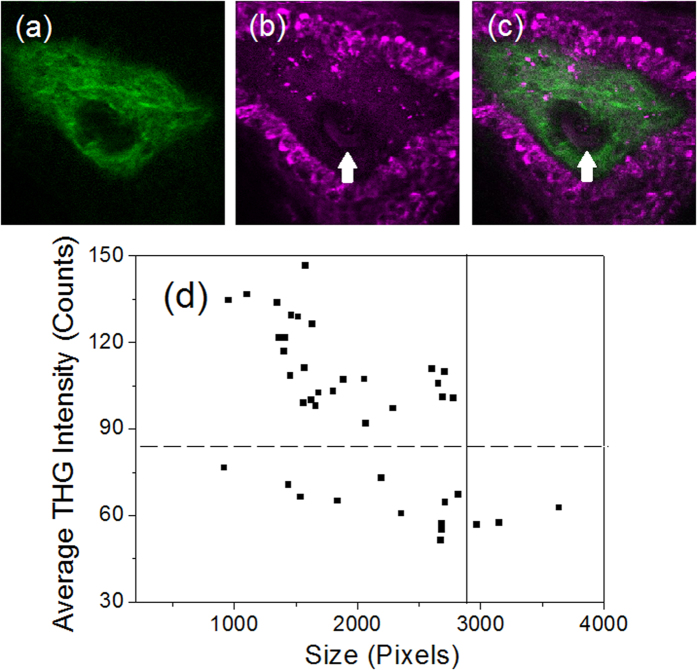
The (**a**) SHG, (**b**) THG and (**c**) combined images of a capillary (indicated by a white arrow) in the dermal papilla region beneath the human skin. The papilla is surrounded by basal cells with strong THG contrast. (**d**) The scatter plot of the average THG intensity and size of WBCs’ cross-sectional images captured in the human capillary (See Fig. S9).

As a feasibility test, we took the *in vivo* THG images of circulating blood cells (Video 1) in the dermal papilla region [green color in Fig. 6(a,c)] beneath human skin. The papilla is surrounded by basal cells and epidermal cells with strong THG contrast [magenta color in Fig. 6(b,c)]. Within the papilla, there is a capillary loop (indicated by white arrows) in this sectioning plane. We took the video-rate *in vivo* THG images on that capillary for 15 minutes and analyzed each flowing blood cells. According to the morphodynamics^9^, sizes and the THG intensities of blood cells, we identified 40 leukocytes (Fig. S9). Analyzing their average THG intensity and cross-sectional area, we found from scatter plot [Fig. 6(d)] that the leukocytes can still be differentiated by setting a threshold of THG intensity (dashed line) and size (solid line) at 84 and 2880, respectively. The cells can be categorized into 26 cells with strong THG intensities, 3 cells with larger cross-sectional areas, and 11 cells with smaller sizes and areas. By assigning them to neutrophils, monocytes, and lymphocytes, respectively, we found the population percentage of each type (Neu: 65%; Mono: 7.5%; Lym: 27.5%) agrees well with the CBC results of the healthy volunteer (Neu: 64.2%; Mono: 6.2%; Lym: 29.6%) at the same date. More clinical trials will be required to determine the specificity and sensitivity of our method.

## Discussion

To avoid the deformation of WBCs on the container’s surface, we limited our THG imaging time within one hour, thus limiting the total number of leukocytes observed. For each type of WBC, we can only manually image 30–60 cells. Due to the variation of the nucleus-to-cytoplasm ratio in the sectioning plane of neutrophils, in some cases, the variance of the average THG intensity was so high that the sensitivity of cell typing was reduced (Fig. S5 and Table S1). This problem can be overcome by motorized imaging acquisition or an imaging flow cytometer, which can increase the number of leukocytes analyzed. The standard error (margin of error) of each parameter could be thus reduced, and the WBCs could be differentiated with higher sensitivity and specificity (Fig. S6). Another solution is to explore different textural features and the underlying nonlinear structure to better distinguish different types of WBCs.

Similar problems were encountered in WBC counting. Because the WBCs were not enriched, under limited fields of view, it took more time to find leukocytes. As a result, only 30–80 cells in total can be counted in the process of THG imaging in the counting chamber (Table 1). Especially for monocytes, one identification error could result in a 2–3% deviation in population percentage. We believe that, with image-guided automatic searching of WBCs, the observation number can be increased and the accuracy of WBC differentials can be greatly improved.

For *in vivo* THG imaging flow cytometry, the result indicates that THG features of leukocytes are still sufficient for the differentiation of leukocyte types *in vivo*. Interestingly, the size of circulating neutrophils is relatively smaller than *in vitro* ones. Besides, the size distributions were broadened, which might be due to the difficulty in maintaining the sectioning plane at its largest cross-sectional area. This problem could be solved by an autofocusing technique based on the alignment of basal cells in the THG images. Another issue is the limitation of imaging frame rate. At 120 Hz frame-rate, to avoid 1 μm image distortion, the lateral flow speed of leukocytes should be lower than 120 μm/sec. According to our previous work, the typical flow speed of blood cells in human capillaries are about 300 μm/sec^21^. To increase the frame rate of imaging, fast tomographic imaging methods like harmonic generation temporal focusing^22^ should be employed. We will develop the system for fast *in vivo* THG imaging in our future works.

In conclusion, based on the label-free and background-free method of THG microscopy, we found that imaging features, such as average intensity and size, were obvious enough for WBC differentiation. Applying a k-means clustering algorithm to the THG intensity/size scatter plot, the WBC types can be differentiated with >95% sensitivity and >97% specificity for isolated cases. Using a differentiation threshold developed from ground-truth THG images, WBCs can be differentiated and counted in a whole blood environment, and the results agree well with those obtained by conventional counting methods. Furthermore, through an *in vivo* THG imaging of circulating blood cells, we found these features can still differentiate leukocyte types and make correct counts. We believe that the current results, including the accuracy of counting with a limited amount of blood, could be further improved by taking textural features and the underlying nonlinear structure into account. Then, the CBC task could be achieved with non-invasive *in vivo* imaging flow cytometry or least invasive *ex vivo* cytometry with fingertip blood.

## Materials and Methods

All the experimental protocols and subject recruitment were approved by the Research Ethics Committee (REC) in National Taiwan University Hospital (NTUH) with permission No. 201203066DIC and also by Taiwan Food and Drug Administration (TFDA). Informed consent was obtained from all subjects. All methods were carried out in accordance with relevant guidelines and regulations.

### Blood collection

Peripheral venous blood (6 mL) was drawn from healthy volunteers by a well-trained clinical scientist in NTUH. Blood was collected in a medical vacutainer (BD Vacutainer) containing anticoagulant (ethylenediamine tetraacetic acid, EDTA) and was kept in the dark at room temperature prior to use. Samples were processed within 1 hour after the sampling of blood.

### Isolation and purification of leukocytes

Two density-gradient separation media, Histopaque-1119 and -1077 (Sigma-Aldrich, Inc.), were used to isolated leukocytes from whole blood. Histopaque-1119 and -1077 and blood samples were added gently into a 15-mL centrifuge tube sequentially in a volume ratio of 1:1:2 and then centrifuged at 900 × *g* and 22 °C for 30 minutes. After centrifugation, granulocytes and agranulocytes were found at the Histopaque−1077/1119 and Histopaque-1077/plasma interfaces, respectively. Each layer was collected with a 15 mL centrifuge tube, the remaining Histopaque was washed out with PBS and enriched by centrifugation at 500 × *g* and 22 °C for 10 minutes. The plasma was diluted to 1% with PBS serving as the cell medium.

After density gradient isolation, a conventional flow cytometer (FACSAria IIIu, BD) was introduced to purify leukocytes. Before sorting, all samples were passed through a cell strainer (352235, BD Falcon) to remove aggregating cells and impurities. By forward scattering (size) and side scattering (granularity) properties, leukocytes were separated into three groups in the scatter plot, which were granulocytes, monocytes and lymphocytes. Anti-FITC antibodies were employed to label and isolate cells more specifically. The cells were enriched by centrifugation at 500 × *g* and 22 °C for 10 minutes. After spinning down, the supernatant of each sample was removed carefully and replaced with 100 μL 1× PBS to re-suspend the cells.

### Histological staining of leukocytes

A blood smear was made firstly by placing a 10-μL blood droplet on one end of a slide. Then, the blood was smeared across the slide, dried, and stained with reagents (Liu’s A and Liu’s B) purchased from Giantech. The Liu’s A was first dripped onto the blood smear. After 30 seconds, the Liu’s B was dripped onto the sample and mixed thoroughly. Finally, the residual dye was washed away with distilled deionized water.

### Immunofluorescent labeling

The immunofluorescent labeling technique was used to isolate specific types of leukocytes in the flow cytometer. Each antibody was conjugated with fluorescein isothiocyanate (FITC), which has excitation/emission wavelengths at 488-/530-nm. Neutrophils and monocytes were labeled with anti-CD66b and anit-CD14, respectively. Lymphocytes were labeled with a mixture of anti-CD3, anti-CD19 and anti-CD56 to isolate all the subtypes of lymphocytes (T-, B- and NK-cell). Cells were stained and kept in dark for 30 minutes. Then, the residual labeling antibodies were washed with PBS, and the cells were enriched by centrifugation at 500 × *g* for 10 minutes. The pellet was re-suspended in 100 μL 1% plasma and then passed through a cell strainer. The cell suspension was sent to the flow cytometer for purification.

For the isolation of specific types of leukocytes, two labeling strategies were used. The “positive labeling” method stained the target leukocytes, while the “negative labeling” method stained all the leukocytes other than the target ones. Using gating by the fluorescence level of FITC, the target cells can thus be isolated with more specificity.

### Mounting of blood cells for THG microscopy

The condenser collecting the transmitted THG signals was a high numerical aperture objective (40×, 1.15 NA) with a short 0.2-mm working distance. Therefore, instead of a thick counting chamber slide, blood cells were sandwiched and mounted in two cover glasses spaced with a thin plastic film. The distance between the two cover glasses was approximately 20 μm, suitable for the mounting of leukocytes (8–15 μm) without squeezing them.

### Clinical complete blood count

After the blood was collected into a 6-mL vacutainer with EDTA, half of the blood was sent to the Department of Laboratory Medicine in NTUH for complete blood counting using an XE-5000 instrument (Sysmex). The rest was diluted 10 times with PBS for cell counting with THG microscopy.

The THG images of each leukocyte within a 2-mm square area were recorded for the analysis of average intensity and size by ImageJ (National Institutes of Health, USA). The cell density was defined as


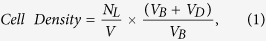


where N_L_ is the number of leukocytes counted, V_B_ is the volume of the original blood, V_D_ is the volume of diluent, and V is the volume of the counting region. The volume V is the distance between the two cover glass slips times the 2-mm-square area.

### Imaging system

The imaging system (Fig. S7) contained a home-built Cr:forsterite femtosecond laser, a scanning unit, an inverted microscope (DMI3000 M, Leica), a water-immersed 63× and 1.15 NA objective (LD C-Apochromat 63x/1.15 W Corr M27, Zeiss), two photomultiplier tubes (H7732–10, HAMAMATSU), and an image acquisition card (Odyssey Xpro, Matrox). The laser was operated at 1250 nm with a 40-nm bandwidth. An additional 488-nm laser (SAPPHIRE 488 LP, Coherent) was combined with the 1250-nm laser beam path to excite the FITC fluorescent dye. The combined excitation beam was reflected by two scanning mirrors and a dichroic mirror (T750DCSPXXR, Chroma Technology). The beam was focused on the sample mounted on a two-dimensional translation stage (H117N1IX/F, Prior). The generated THG signal and the emitted FITC fluorescence signal were collected by a water-immersed objective (UApo N 340 40x/1.15NA W, Olympus) and were split by a long pass dichroic beam splitter edged at 510 nm (T510lpxrxt, Chroma). Both signals were focused onto each PMT for detection. The analog photo currents were converted into voltages by trans-impedance amplifiers (C6438-01, HAMAMATSU) and then digitized by analog-to-digital converters on the image acquisition card. Sampled signals were reconstructed into a 512 × 512 image in software point-by-point and presented on the screen with a 30-Hz frame rate.

### *In-vivo* THG imaging cytometry

The healthy volunteer put his forearm on the stage of an inverted microscope. A cover glass was mount on the stage in order to make sure the skin was flattened. The laser power after the objective was kept below 130 mW. While acquiring the THG images, the focal plane was set at the junction of epidermis and dermis, where the capillaries were located. The SHG and THG signals were epi-collected by the same objective (UApo N 340 40x/1.15NA W, Olympus) and were split by a long pass dichroic beam splitter edged at 510 nm (T510lpxrxt, Chroma). The scheme of PMT detection, amplifiers, and data acquisition are similar to the *in vitro* system. The experimental cycle includes a 3-minute laser exposure time and a 10-second break. Total *in vivo* imaging time was around 15 minutes. By the end of the clinical trial, a 3-ml blood from the volunteer was sampled by clinical scientist and was analyzed by clinical complete blood count in NTUH afterward.

## Additional Information

**How to cite this article**: Wu, C.-H. *et al*. Imaging Cytometry of Human Leukocytes with Third Harmonic Generation Microscopy. *Sci. Rep*. **6**, 37210; doi: 10.1038/srep37210 (2016).

**Publisher’s note:** Springer Nature remains neutral with regard to jurisdictional claims in published maps and institutional affiliations.

## Supplementary Material

Supplementary Information

Supplementary Video S1

## Figures and Tables

**Table 1:** Counting number (N), percentage and cell density from THG microscopy compared with CBC test.

	THG Microscopy					CBC				
	N	Neu (%)	Mono (%)	Lym (%)	Cells/μL	N (×10^5^)	Neu (%)	Mono (%)	Lym (%)	Cells/μL
1	33	69.7	3	27.3	4940	2.14	69.4	7.4	23.2	5350
2	48	68.9	8.9	22.2	5925	2.768	65.9	10.7	23.4	6920
3	81	69.5	4.9	25.6	10008	3.268	65.1	6.6	28.8	8170
4	50	70.4	7.4	22.2	9182	2.06	62.3	6.8	30.9	5150
5	34	51.7	13.8	34.4	7927	2.648	53.9	7.6	38.5	6620

Neu: neutrophils; Mono: monocytes; Lym: lymphocytes.
