# Supplementary material for: Imaging Cytometry of Human Leukocytes with Third Harmonic Generation Microscopy
Source: Sci Rep. 2016 Nov 15;6:37210. doi: 10.1038/srep37210 (PMC5109028; doi:10.1038/srep37210)
Supplement: Supplementary Information [file srep37210-s1.pdf]

Supplementary Information

Imaging Cytometry of Human Leukocytes with Third Harmonic Generation Microscopy

Cheng-Han Wu[†], Tzung-Dau Wang[§],Chia-Hung Hsieh[†], Shih-Hung Huang[†], Jong-Wei Lin[§], Szu-Chun Hsu[#], Hau-Tieng Wu[&], Yao-Ming Wu[¶], and Tzu-Ming Liu[ǀ,†,‡]

[†]Institute of Biomedical Engineering, National Taiwan University, Taipei 10617, Taiwan
[§]Cardiovascular Center and Division of Cardiology, Department of Internal Medicine, National Taiwan University Hospital and College of Medicine, Taipei 10002, Taiwan
[#]Department of Laboratory Medicine, National Taiwan University Hospital, Taipei 10002, Taiwan
[&]Department of Mathematics, University of Toronto, Toronto, Canada
[¶]Department of Surgery, National Taiwan University Hospital and College of Medicine, Taipei 10002, Taiwan
[‡]Molecular Imaging Center, National Taiwan University, Taipei 10617, Taiwan
[ǀ]Faculty of Health Sciences, University of Macau, Macao SAR, China

Comparison of THG images with hematology stains

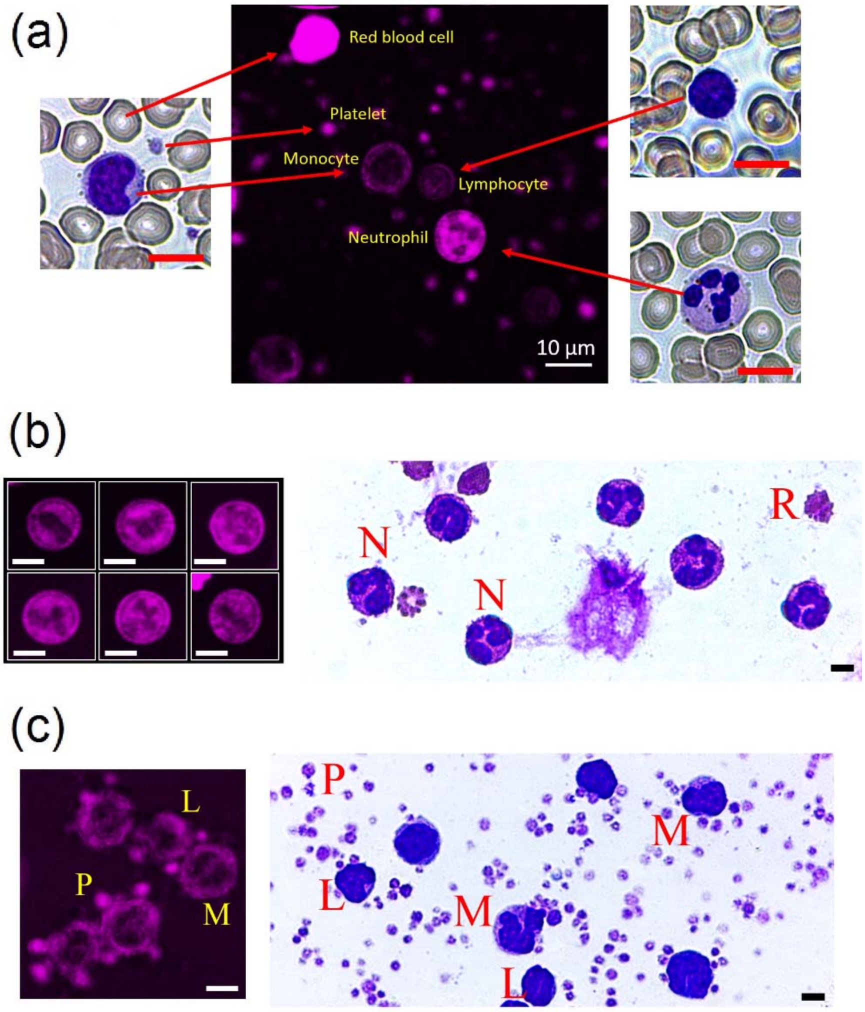

Figure S1. Comparison of the THG sectioning images (magenta color) with the

hematology pictures using Liu's stain on (a) the whole blood smear, (b) the isolated granulocyte band and (c) non-granulocyte band from Histopaque gradient centrifugation. R: red blood cells; N: neutrophils; L: lymphocyte; M: monocyte; P: platelet. Scale bars: (a) 10 µm and (b) (c) 5 µm.

Anticoagulant effects

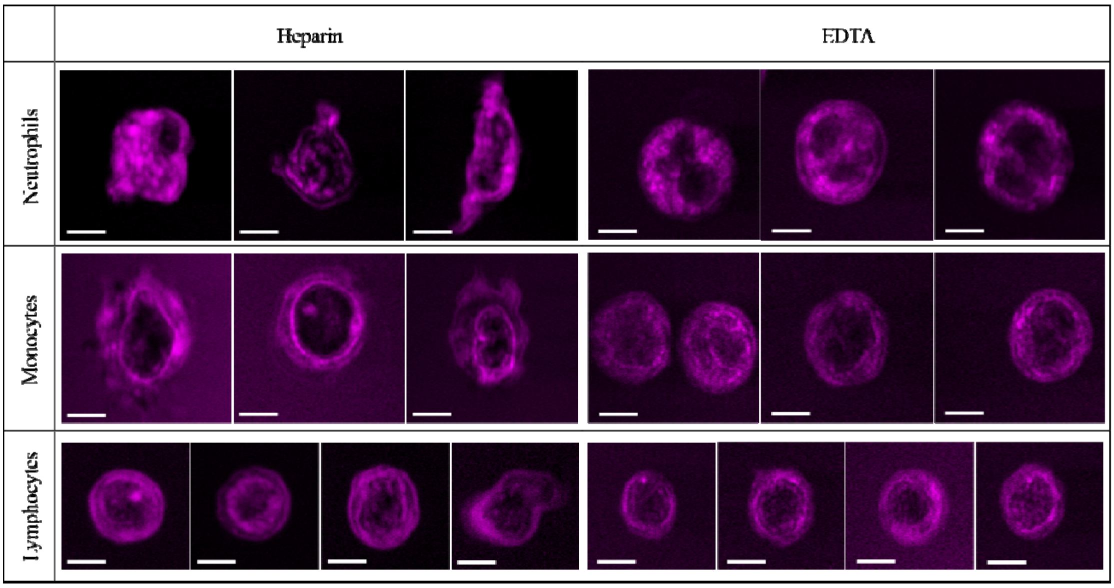

Figure S2. Comparison of the THG sectioning images of various leukocytes from the heparin-coated tube and EDTA-coated tube. Scale bars: 5 µm.

Surface contact effects

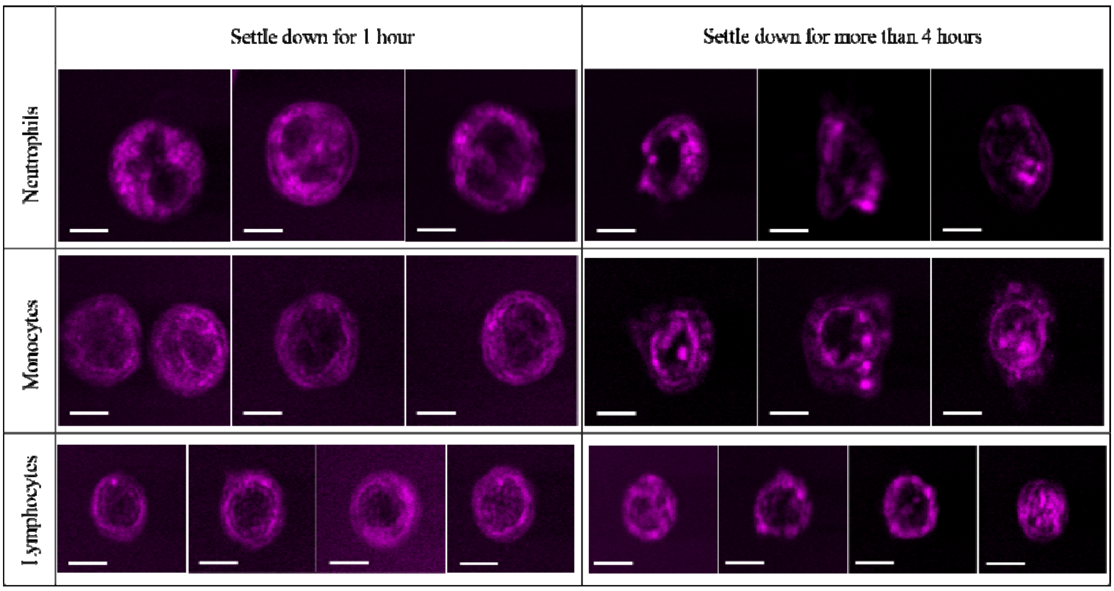

Figure S3. Comparison of the THG sectioning images of various leukocytes settled down for 1 hour and more than 4 hours. Scale bars: 5 µm.

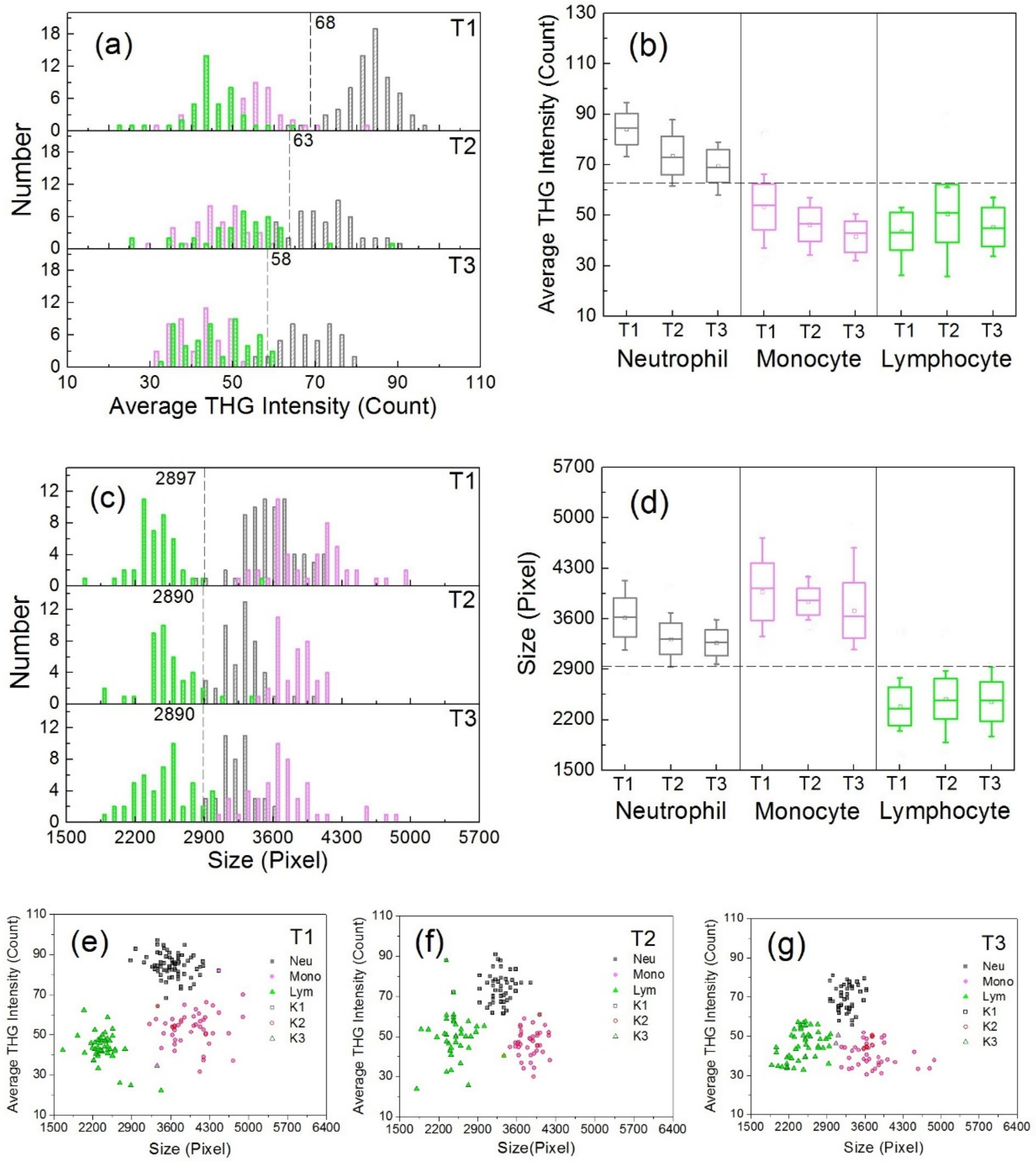

Figure S4. (a,c) Histograms, (b,d) box charts, and (e,f, g) scatter plots of (a,b) the average THG intensity and (c,d) size of WBC. The neutrophils (gray), monocytes (light magenta), and lymphocytes (green) were collected from the volunteer 1 at three different time points T1, T2, and T3. In the box chart, box range cover the mean ± standard deviation and the whiskers cover 5% to 95% of the data. Three groups of data K1 (black open square), K2 (open red circle), and K3 (open olive triangle) were clustered by the k-means clustering analysis. Neu: neutrophils; Mono: monocytes; Lym: lymphocytes.

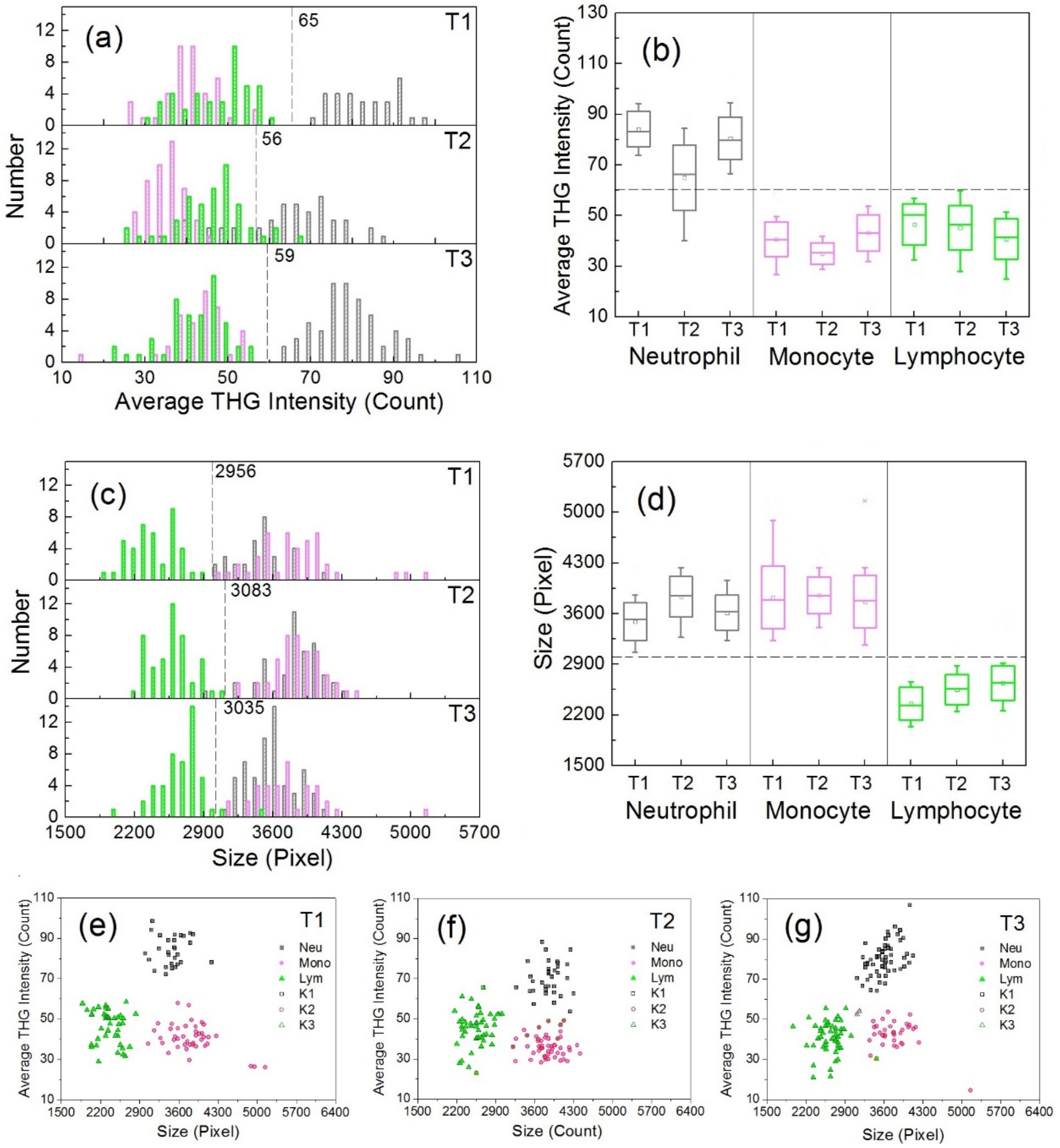

Figure S5. (a,c) Histograms, (b,d) box charts, and (e,f, g) scatter plots of (a,b) the average THG intensity and (c,d) size of WBC. The neutrophils (gray), monocytes (light magenta), and lymphocytes (green) were collected from the volunteer 2 at three different time points T1, T2, and T3. In the box chart, box range cover the mean ± standard deviation and the whiskers cover 5% to 95% of the data. Three groups of data K1 (black open square), K2 (open red circle), and K3 (open olive triangle) were clustered by the k-means clustering analysis. Neu: neutrophils; Mono: monocytes; Lym: lymphocytes.

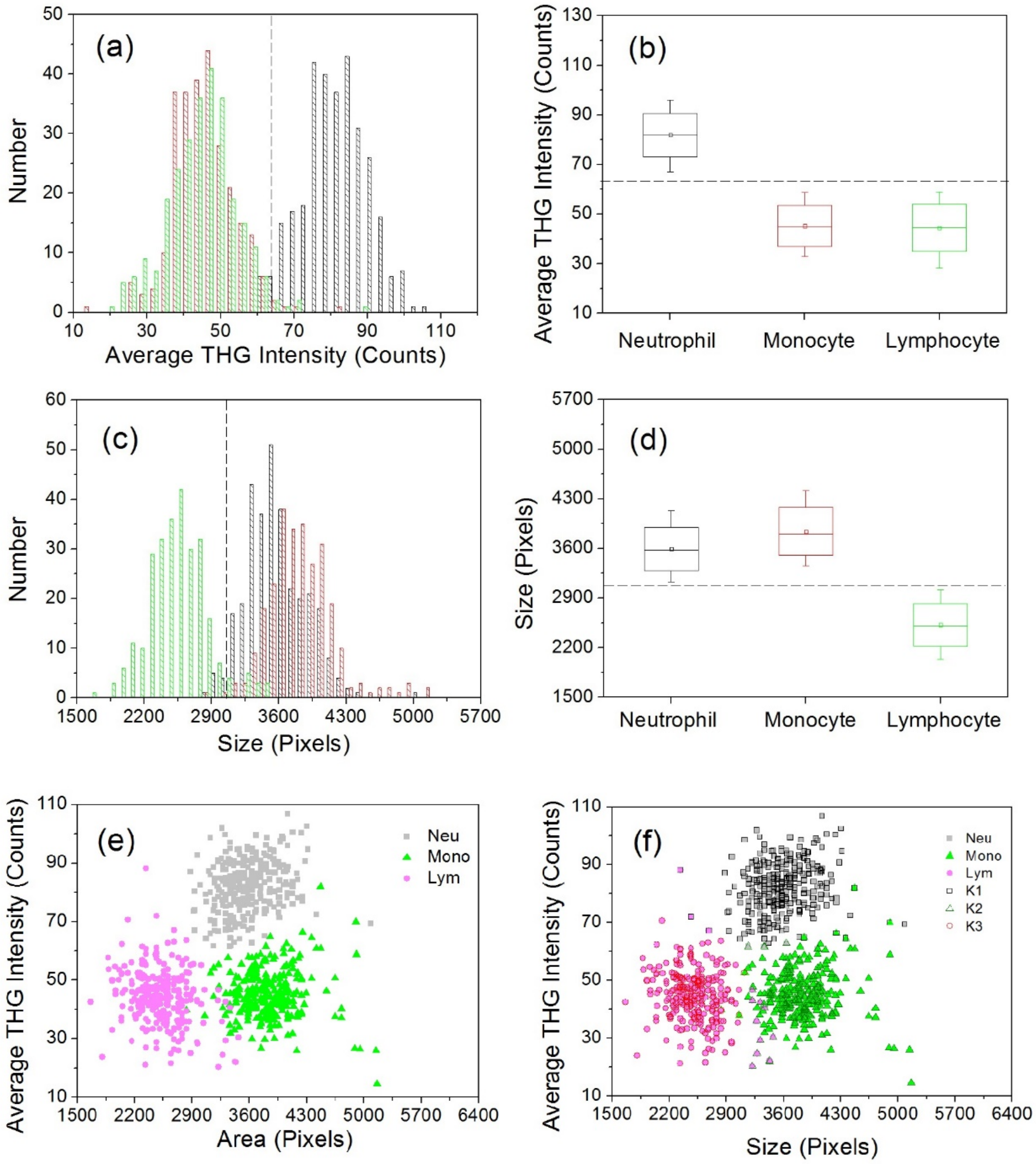

Figure S6. (a,c) Histograms, (b,d) box charts, and (e,f) scatter plots of (a,b) the average THG intensity and (c,d) size of the WBC. The data points of neutrophils (gray), monocytes (light magenta), and lymphocytes (green) from all three healthy volunteers were put together. In the box chart, box ranges cover the mean ± standard deviation, and the whiskers cover 5% to 95% of the data. Three groups of data K1 (black open square), K2 (open red circle), and K3 (open olive triangle) were categorized by the k-means clustering analysis. Neu: neutrophils; Mono: monocytes; Lym: lymphocytes.

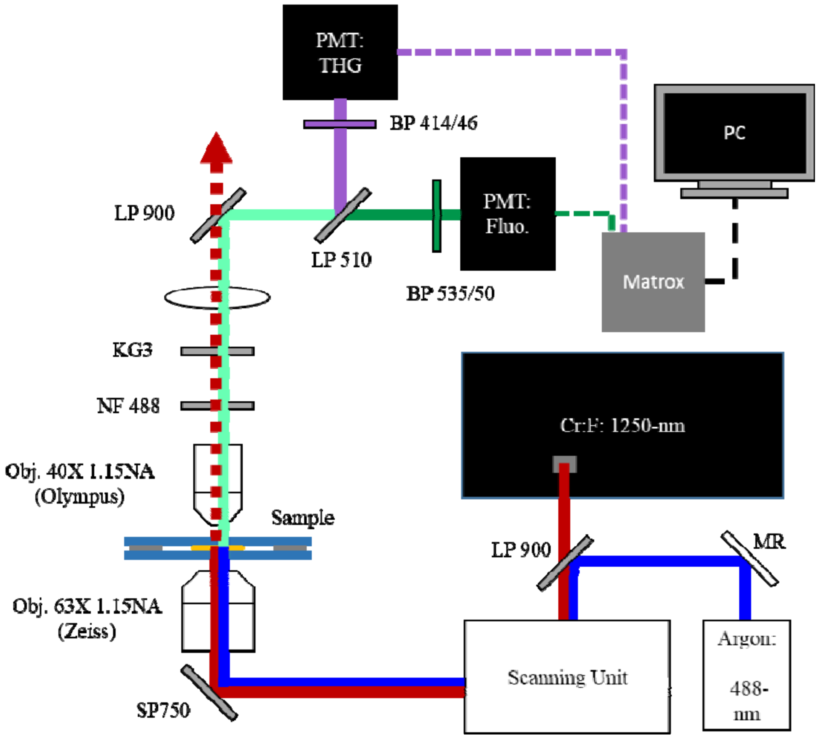

Figure S7. Setup of the THG Imaging system. MR: silver mirror (PF10-03-P01, Thorlabs); LP 900: long pass dichroic beamsplitter edged at 900-nm (DMLP900R, Thorlabs); SP 750: short pass dichroic beamsplitter edged at 750-nm (T750DCSPXXR, Chroma Technology); Obj: Objective; NF 488: notch filter for 488-nm laser line (NF03-488E-25, Semrock); KG3: colored glass filter with high transmission at 315-710 nm (FGS900, Thorlabs); LP 510: long pass dichroic beamsplitter edged at 510-nm (T510lpxrxt, Chroma); BP 414/18: bandpass filter edged at 414-nm (FF01-414/46-25, Semrock); BP 535/50: bandpass filter edged at 535-nm (FF01-535/50, Semrock); PMT: photomultiplier tubes; PC: personal computer; Matrox: image acquisition card.

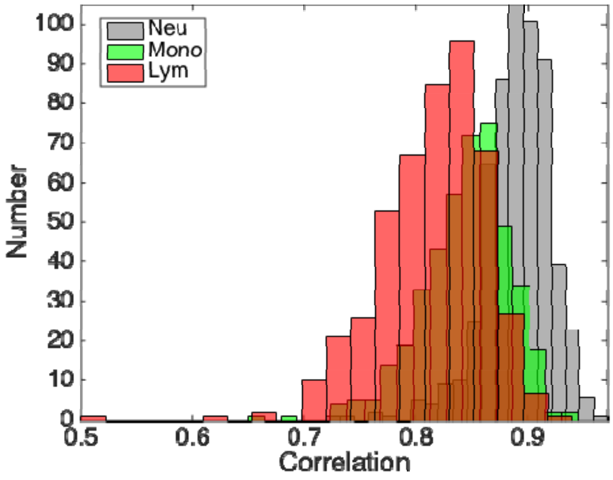

Figure S8. The histogram of three WBC types from three healthy subjects with the texture feature "correlation" is shown. The texture feature is determined from the co-occurrence matrix based on the Haralick definition. The correlation coefficient between the correlation feature and the mean intensity is 0.61, and the correlation coefficient between the correlation feature and the volume is 0.4. The mean and standard deviation of neutrophil, monocyte, and lymphocyte are 0.89±0.03, 0.85±0.04, and 0.82±0.05. The distribution of each WBC types is significantly different from the others, determined by the Mann-Whitney U test with the p-value less than 10^{-4}. In particular, the texture feature provides a distinguishable feature for the monocyte and neutrophil.

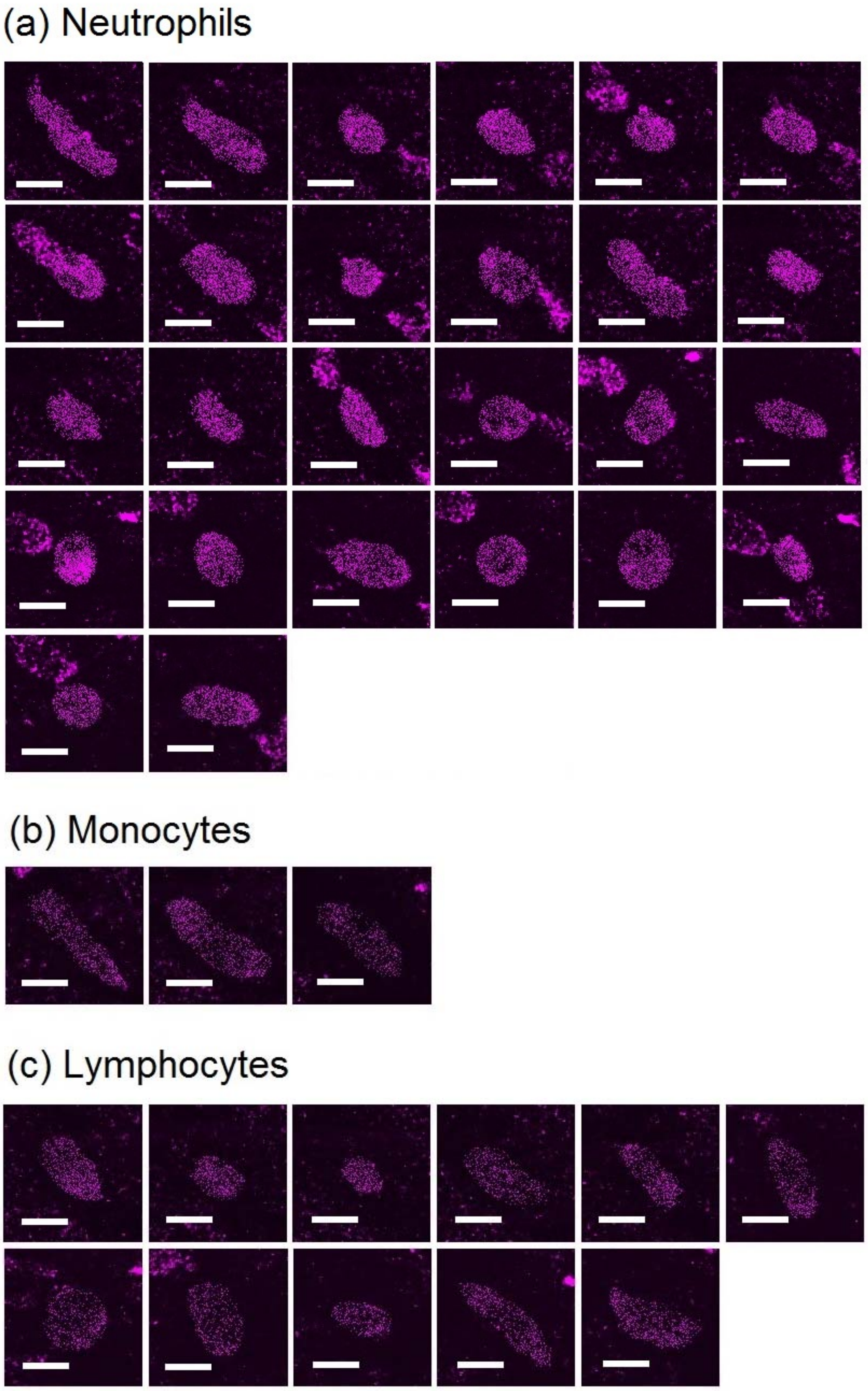

Figure S9. The *in vivo* THG images of human leukocytes captured from the dermal capillary of a healthy volunteer. According to the thresholds of THG intensity and size plotted in Figure 6, the cells can be categorized into (a) neutrophils, (b) monocytes and (c) lymphocytes. Scale bars: 5 μm.

Volunteer	Time	WBC	TP	TN	FP	FN	Sensitivity	Specificity
V1	T1	N	70	92	1	1	99%	99%
		M	47	114	1	2	96%	99%
		L	44	119	1	0	100%	99%
	T2	N	47	77	2	1	98%	97%
		M	39	86	2	0	100%	98%
		L	37	87	0	3	93%	100%
	T3	N	44	99	0	0	100%	100%
		M	47	94	0	2	96%	100%
		L	52	89	2	0	100%	98%
V2	T1	N	30	83	0	0	100%	100%
		M	42	71	0	0	100%	100%
		L	41	72	0	0	100%	100%
	T2	N	35	92	1	9	80%	99%
		M	46	82	9	0	100%	90%
		L	45	90	1	1	98%	99%
	T3	N	59	84	0	0	100%	100%
		M	34	106	1	2	94%	99%
		L	47	93	2	1	98%	98%
V3	T1	N	41	87	2	1	98%	98%
		M	44	82	4	1	98%	95%
		L	39	86	1	5	89%	99%

Table S1. Calculating the sensitivity and specificity of identifying a certain type of WBC with k-means clustering algorithm. Seven batches of data were obtained from three volunteers, V1, V2, and V3 at seven different time points. N: neutrophils; M: monocytes; L: lymphocytes; TP: true positive; TN: true negative; FP: false positive; FN: false negative.

Movie captions

The movie of THG sectioning images in the capillary of a dermal papilla. Frame rate: 5 Hz. Field of view: 85 × 85 μm.
