# Supplementary figures and images for: Imaging Cytometry of Human Leukocytes with Third Harmonic Generation Microscopy
Source: Sci Rep. 2016 Nov 15;6:37210. doi: 10.1038/srep37210 (PMC5109028; doi:10.1038/srep37210)

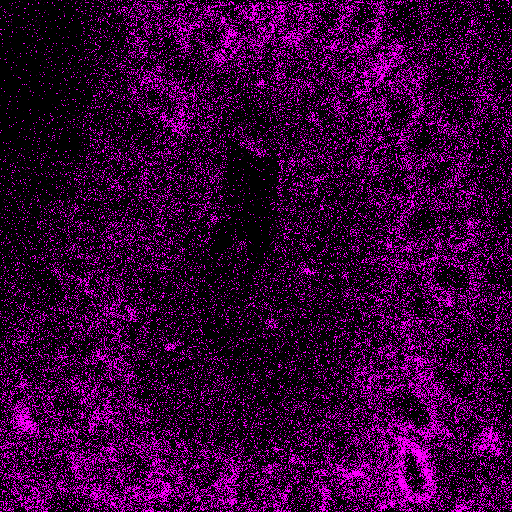

Supplement: Supplementary Video S1 [file srep37210-s2.gif]
